# Mood changes at very high altitudes in Pakistan

**DOI:** 10.12669/pjms.331.11393

**Published:** 2017

**Authors:** Sabih Ahmad, Sadiq Hussain

**Affiliations:** 1 Dr. Sabih Ahmad, FCPS. Department of Psychiatry, Consultant Psychiatrist, Combined Military Hospital, Gilgit, Pakistan; 2 Dr. Sadiq Hussain, PhD. Department of Behavioral Sciences, Head of Department, Karakorum International University, Gilgit, Pakistan

**Keywords:** Anxiety, Altitude, Depression, Hypoxia, Mood

## Abstract

**Objective:**

To screen out psychiatric ‘cases’ and find the frequency of anxiety and depression symptoms in military volunteers performing duties at very high altitudes in the Karakoram ranges of Pakistan.

**Methods:**

This was a descriptive study lasting from Jan 2015 to June 2015, on volunteers serving at very high altitude, using General Health Questionnaire-12 (GHQ-12) and Hospital Anxiety and Depression Scale (HADS), Urdu versions. Analysis involved descriptive, inferential techniques and Bonferroni test. Demographic variables were compared to the scores.

**Results:**

A high percentage of the military volunteers screened positive for psychiatric ‘caseness’ and symptoms of anxiety and depression; mostly in the mild to moderate range, while very few of them reported severe symptoms. Demographic variables such as marital status, number of children, positive family psychiatric history, past medical history, duration at high altitude and educational levels were found to be significant risk factors for developing symptoms of anxiety and depression.

**Conclusions:**

Individuals performing duties at very high altitude, experience symptoms of anxiety and depression, their demographics are important in understanding their emotional problems.

## INTRODUCTION

The efficiency to perform of an individual can increase or decrease with the change in his mood. This becomes more evident especially when he cannot be in charge of his environmental dynamics.^1^

The very high altitudes of the Karakoram mountain ranges in Gilgit Baltistan region of Pakistan are manned by troops and volunteers, as high as 24,000 feet above mean sea level (AMSL). Such altitudes have long been postulated to cause changes in human behavior. Some sporadic literature is available that is widely spread over many years, attempting to describe the psychiatric point of view at such difficult terrains. Most literature available is based on findings in mountaineers, sportsmen or animals, and that too mostly in simulated environments.^2^ Very little information is available on the military personnel, while deployed ‘up there’.

As per the US Army medical department’s^3^ classification, we conducted our study at Very high altitude, in classic hypobaric hypoxic conditions.^4^ Changes in neurotransmitter synthesis have been proposed as an underlying cause of these altered moods and behavior.^5^ A simulated study at mild to very high hypoxic environment ascent for one hour each, revealed ‘adverse affects’ on moods that increased with increased hypoxia.^6^

Various psychiatric changes at high altitudes have been described earlier^6^,^7^ with most available work emphasizing on increased anxiety.^8^,^9^ Behavioral impairments caused by ascent to high altitudes can thus affect various operations, since both rate of performance and judgment are affected.^8^,^10^

An additional factor here was the adverse living conditions at these heights. These may include poor nutrition, lack of exercise, poor hygiene, wilderness, solitude and a lot more. Our objective was to find out whether any described mood changes occur in our courageous volunteers serving at very high altitude, and to what extent. This could be a first step at developing newer protocols for their mental well being.

## METHODS

A total of 129 military participants took part in this five month long descriptive study, at a height of between 15000 feet AMSL to 18000 feet AMSL, at the Karakorum ranges in the Gilgit Baltistan region of Pakistan. Author SA, a consultant psychiatrist, climbed to the very high altitude himself, undergoing all acclimatization protocols, and interviewed all the participants at their place/height of deployment. Written consent was obtained from all volunteers after explaining the purpose of the current study. We included all personnel, deployed at a very high altitude based on purposive sampling technique. Those with past or present history of psychiatric illness, history of psychotropic drug use, or a current history of any general medical condition, were excluded along with those who descended down after completing their duties to ground level/lower height.

A proforma was developed including demographics, period on the height, past medical history, family history of psychiatric or medical ailments, and history of serving at high altitude earlier in the last one year.

For screening psychiatric cases we used General Health Questionnaire - 12, Urdu version (GHQ-12)^11^ which is a validated screening tool for ‘cases’ in general population, in terms of anxiety, depression, insomnia and social dysfunction, having a high sensitivity and specificity in similar populations.^12^

The second instrument used in our study was Hospital Anxiety and Depression Scale (HADS), which is a reliable instrument for detecting and rating anxiety and depression symptoms in hospitalized patients^13^ with a high sensitivity and specificity,^14^ but has been utilized in other situations too in Pakistan, where it remained one of the very few such instruments available in Urdu.^15^ Anxiety and depression were both categorized into ‘mild’, ‘moderate’ and ‘severe’.

The Institutional Ethical Review Committee approved our research proposal. For statistical analysis of collected data, we used descriptive (mean, standard deviation & frequencies) and, inferential (t-test, Pearson product moment correlation, & analysis of variance) statistical techniques and Bonferroni test for post-hoc analysis, in Statistical Package for Social Sciences (SPSS version 21).

## RESULTS

Majority of research participants were married (51%) with an age ranging from 18 to 41 years (*M=28.1, SD=6.5*). Most of the participants (37%) had ten years of schooling, 16% reported having 14 years of schooling, and only 2% were illiterate. Fifty six percent participants reported a past history of performing similar duties at high altitudes.

According to our findings with the screening tool for psychiatric ‘cases’ (GHQ-12), majority of the participants reported having psychiatric problems (Table-I & II).

**Table-I:** Mean and standard deviation on HADS and GHQ-12 scale (N=129).

Variables	N	M	SD
HADS Depression	129	6.91	3.9
HADS Anxiety	129	7.93	5.1
GHQ-12	129	4.93	3.1

**Table-II:** Range, frequency, and interpretation of participants’ scores on HADS and GHQ-12 scale (N=129).

Variable	Range	Frequency	Percentage	Interpretation
HADS (Depression Scale)	8-10	27	21%	Mild depression
	11-14	15	12%	Moderate depression
	15-21	9	7%	Severe depression
HADS (Anxiety Scale)	8-10	27	21%	Mild anxiety
	11-14	27	21%	Moderate anxiety
	15-21	6	5%	Severe anxiety
GHQ-12	3 and above	96	74%	‘Cases’

Therefore, to rule out any clinically significant level of depression and anxiety we used a second tool (HADS). The relationships between the two scales; HADS anxiety (*r=.36, p=.00*) and depression portion and GHQ-12 (*r=.7, p=.00*) were significant and both anxiety and depression were also significantly correlated (*r=.26, p=.00*). For further clarification of our results, individual scores were assessed by computing frequencies (Table-II).

We evaluated the role of demographic variables such as marital status, prior history of performing duties at very high altitudes in the last one year, duration of stay at high altitude, age, qualification, positive psychiatric history in the first degree relatives, past medical history in the last one year, and number of children the individual had in the current level of anxiety and depression. Results indicated that there were no significant differences in the levels of anxiety and depression between married and unmarried military personnel, fist time deployed personnel, and those who had performed their duties at very high altitude earlier in the preceding year. But on GHQ-12 analysis, married volunteers reported significantly higher level of psychiatric problems as compared to unmarried volunteers (Table-III).

**Table-III:** Level of anxiety and depression between married and unmarried volunteers and with and without prior history of serving at very high altitude.

Scales	Demographics	N	M	SD	t	p
HADS (Depression Scale)	Married	66	7.5	3.1	1.8	.07
	Unmarried	60	6.3	4.7		
HADS (Anxiety scale)	Married	66	7.2	4.1	1.7	.08
	Unmarried	60	8.8	6.03		
GHQ-12	Married	66	5.8	3.1	3.03	.01**
	Unmarried	60	4.1	2.9		
HADS (Depression Scale)	Newly Deployed	57	7.4	4.9	1.3	.19
	Have Past History	72	6.5	3.0		
HADS (Anxiety scale)	Newly Deployed	57	7.0	4.6	1.7	.08
	Have Past history	72	8.6	5.5		
GHQ-12	Newly Deployed	57	4.7	3.4	.6	.5
	Have Past history	72	5.0	2.9		

** p<.01

There was a significant and negative correlation (*r=-.22, p=.01*) between duration and level of anxiety among the volunteers. It points towards the finding that when duration at very high altitude increased, their level of anxiety decreased. There was also a significant and negative correlation (*r=-.26, p=.00*) between number of children and the level of anxiety. It was seen that as the number of children increased the volunteers’ level of anxiety decreased. Participants’ educational level also came out as a significant variable in explaining their level of anxiety at very high altitudes (Table-IV). For further clarification of these findings post-hoc analysis was performed.

**Table-IV:** Analysis of variance results levels of anxiety and depression as dependent variable while their educational levels as independent variable.

Variable	Sources	df	SS	MS	F
HADS (Depression Scale)	Between Groups	5	168.5	33.7	2.2
	Within Groups	123	1864.3	15.1	
	Total	128	2032.8		
HADS (Anxiety Scale)	Between Groups	5	780.6	156.1	7.3**
	Within Groups	123	2611.7	21.2	
	Total	128	3392.3		
GHQ-12	Between Groups	5	171.1	34.2	3.7**
	Within Groups	123	1109.2	9.01	
	Total	128	1280.3		

** p<.01

According to the findings of post-hoc analysis, volunteers with 12 years of schooling reported higher levels of anxiety as compared to the volunteers with 10 and 8 years of schooling. The volunteers with 10 years of schooling, reported higher level of anxiety as compared to volunteers with 8 years of schooling, and volunteers with five years of schooling reported significantly higher level of anxiety as compared to the volunteers who had never gone to school.

Regarding the role of positive family psychiatric history and past medical history in current level of anxiety and depression: only six of the volunteers reported having a positive psychiatric family history and they reported higher level of depression (*M=12.5*) and anxiety (*M=11*). Six volunteers reported a positive past medical history in the last one year, and they reported significant level of anxiety (*M=9*) too, but their level of depression (*M=5*) was not significant.

We also assessed the most commonly occurring symptoms of anxiety and depression. Findings showed that on HADS, the most commonly occurring symptoms of anxiety were: ‘mental strain’ and ‘feelings of restlessness’ and of depression were ‘inability to enjoy things’. On GHQ-12, the most commonly occurring symptom was ‘an inability to enjoy things’; consistent with the findings on the HADS depression scale.

Findings of current study implied that the military personnel performing their duties at very high altitude reported significant levels of anxiety and depression symptoms regardless of their prior history of performing duties at such areas. Furthermore, their marital status, duration at high altitude, positive family psychiatric history, past medical history, and education level tend to influence their reported level of current anxiety.

## DISCUSSION

In the past there is no study available in the region that has taken a cohort at the height of, and during the military personnel’s deployment, except for the one that assessed changes in cognitive state at high altitude.^4^

The instruments we used gave us significant psychiatric symptoms. Similar findings were presented by past researches from China and USA.^6^,^7^,^16^ Although these studies did not show a very consistent range of positive psychiatric symptoms, they repeatedly emphasized on the presence of anxiety and depression symptoms. The comparable findings give us a figure of as big as 98% subjects suffering from anxiety symptoms during their high altitude stay to as low as 0.67%.^8^,^16^ Our findings (47%) lie somewhere in the middle of the previous findings. Then there were other disparities between our study and the ones by Fagenholz^9^ and Dong.^10^ These included different length of stay, different heights, lower or higher, and different demographical characteristics of the subjects i.e. studies including females, sportsmen, non-deployed military personnel on training, and not collecting the data at the height of deployment, with the assessor not present at the same height as the volunteers. The anxiety symptoms in our cohort were mostly in the mild to moderate range.

Depressive symptoms in our study (40%), were almost comparable to a previous study in the same settings, i.e. 37.8%.^13^ And these were mostly in the mild range. It is pertinent to note that on screening the volunteers with GHQ-12, 74% were marked as cases, but on administering HADS they were found to be having mild to moderate anxiety or depression symptoms at the most.

The role of other variables like schooling, number of children previous history of ascent to high altitude, marital status of the volunteer, a family history of psychiatric illness, were however not found in any earlier studies. Some previous studies show an increase in psychiatric symptoms with the increase in length of stay or increasing ascent in height,^17^ while ours on the contrary showed that the psychiatric symptoms decreased as the time at high altitude deployment increased.

Population with a particular years of schooling and marital status have been found to be prone to develop psychiatric disorders in a previous study by Akhtar-Danesh.^18^ It was shown that not being married increased the probability of getting depression, contrary to our findings. But our findings were related to high altitude unlike the quoted study. However the relationship of having a particular number of children and developing depression or anxiety was not found on literature search by the authors. Similarly first degree relatives having some psychiatric illnesses have shown to make individuals more prone to develop similar issues,^19^ findings similar to ours, but not in relation to high altitude.

The aim of our study never was to diagnose psychiatric syndromes. We however found significant symptoms of anxiety and depression that may or may not have led to a psychiatric diagnosis.

The present study has a novel value by being a first of its kind where data was collected at the height of the deployment of military personnel, instead of waiting for them to descend on lower heights or base camps. It intended on giving a better understanding on the psychological state of the troops deployed at very high altitude, and could be the watershed study for developing psychological measures to further enhance the capabilities of our troops deployed at such difficult heights.

### Limitations and Areas for Future Work

In the future a control group, psychosis, a larger sample size and follow up need to be considered.

## CONCLUSION

The individuals performing duties at very high altitudes, experience symptoms of anxiety and depression. Most prominent areas of disturbance, mostly in the mild and moderate range, were an inability to enjoy daily routines, restlessness and mental strain.

## References

[1] de Aquino Lemos V, Antunes HK, dos Santos RV, Lira FS, Tufik S, de Mello MT (2012). High altitude exposure impairs sleep patterns, mood, and cognitive functions. Psychophysiology.

[2] Bahrke MS, Shukitt-Hale B (1993). Effects of altitude on mood, behavior and cognitive functioning. A review. Sports Med.

[3] Altitude Effects on the Human Body - Army Public Health Center [Internet] http://phc.amedd.army.mil/topics/discond/ai/Pages/AltitudeEffects.aspx.

[4] Ahmed S (2011). Changes in the cognitive state in hypobaric hypoxic condition in Pakistan. Pak Armed Forces Med J.

[5] Kumar GK (2011). Hypoxia Hypoxia and neurotransmitter synthesis. Am J Physiol Cell Physiol.

[6] Li XY, Wu XY, Fu C, Shen XF, Wu YH, Wang T (2000). Effects of acute mild and moderate hypoxia on human mood state. Space Med Med Eng (Beijing).

[7] Shukitt BL, Banderet LE (1988). Mood states at 1600 and 4300 meters terrestrial altitude. Aviat Space Environ Med.

[8] Oliver SJ, Sanders SJ, Williams CJ, Smith ZA, Lloyd-Davies E, Robert R (2012). Physiological and psychological illness symptoms at high altitude and their relationship with acute mountain sickness: a prospective cohort study. J Travel Med.

[9] Fagenholz PJ, Murray AF, Gutman JA, Findley JK, Harris NS (2007). New-onset anxiety disorders at high altitude. Wilderness Environ Med.

[10] Dong JQ, Zhang JH, Qin J, Li QN, Huang W, Gao XB (2013). Anxiety correlates with somatic symptoms and sleep status at high altitudes. Physiol Behav.

[11] Minhas F, Mubbashar MH (1996). Validation of General Health Questionnaire (GHQ-12) in primary care settings of Pakistan. J Coll Physic Surg Pak.

[12] Bashir K (2008). Psychiatric morbidity amongst the troops deployed at Siachen. Pak Armed Forces Med J.

[13] Zigmond AS, Snaith RP (1983). The hospital anxiety and depression scale. Acta Psychiatr Scand.

[14] Mumford DB, Tareen IA, Bajwa MA, Bhatti MR, Karim R (1991). The translation and evaluation of an Urdu version of the Hospital Anxiety and Depression Scale. Acta Psychiatr Scand.

[15] Bjelland I, Dahl AA, Haug TT, Neckelmann D (2002). The validity of the Hospital Anxiety and Depression Scale. An updated literature review. J Psychosom Res.

[16] Sracic MK, Thomas D, Pate A, Norris J, Norman M, Gertsch JH (2014). Syndrome of acute anxiety among marines after recent arrival at high altitude. Mil Med.

[17] Zhang G, Zhou SM, Yuan C, Tian HJ, Li P, Gao YQ (2013). The effects of short-term and long-term exposure to a high altitude hypoxic environment on neurobehavioral function. High Alt Med Biol.

[18] Akhtar-Danesh N, Landeen J (2007). Relation between depression and sociodemographic factors. Int J Ment Health Syst.

[19] Savitz J, Nugent AC, Bellgowan PSF, Wright N, Tinsley R, Zarate CA (2013). Catecholamine depletion in first-degree relatives of individuals with mood disorders: An [(18)F] fluorodeoxyglucose positron emission tomography study. Neuroimage Clin.

